# Psychophysiological and Performance Effects of Biofeedback and Neurofeedback Interventions in a Top 100 Female Chess Player

**DOI:** 10.3390/bs14111044

**Published:** 2024-11-05

**Authors:** Juan Pedro Fuentes-García, Santos Villafaina

**Affiliations:** 1 Grupo de Investigación Análisis Didáctico y Comportamental del Deporte (ADICODE), Departamento de Didáctica de la Expresión Musical, Plástica y Corporal, Facultad de Ciencias del Deporte, Universidad de Extremadura, Avenida de la Universidad s/n, 10003 Cáceres, Spain; 2 Instituto Universitario de Investigación e Innovación en el Deporte, Universidad de Extremadura, 10003 Cáceres, Spain; 3 Grupo de Investigación en Actividad Física, Calidad de Vida y Salud (AFYCAV), Departamento de Didáctica de la Expresión Musical, Plástica y Corporal, Facultad de Ciencias del Deporte, Universidad de Extremadura, Avenida de la Universidad s/n, 10003 Cáceres, Spain

**Keywords:** chess, anxiety, autonomic modulation, performance, problem-solving

## Abstract

(1) Background: Previous studies showed that neurofeedback and biofeedback could improve stress levels, enhance self-control over physiological factors, improve behavioral efficiency, and increase reaction speed to stimuli. Specifically, the sensorimotor rhythm stimulation (12–15 Hz) can enhance cognitive functions such as selective attention and working memory. However, there is no study that analyzes the effect of these interventions in chess players. (2) Methods: A Chess Woman Grandmaster and Chess International Master, with an ELO ranking higher than 2350 points, was selected to participate in this case study. The participant conducted a total of 14 sessions of biofeedback and neurofeedback, training in breathing, sensorimotor rhythm stimulation in Cz, skin conductance, temperature, and heart rate variability combined with chess work. Specific and non-specific tasks were designed to evaluate the intervention. (3) Results: The chess player enhanced the heart rate variability during specific and non-specific chess tasks: chess problems, 15 + 10 games, and puzzle rush games. In addition, the sensorimotor rhythm power decreased during the chess problem and increased during the 15 + 10 game and puzzle rush. Also, chess performance and anxiety levels improved after the intervention. (4) Conclusions: Neurofeedback and biofeedback training combined with chess training could improve the performance of chess players.

## 1. Introduction

Chess is a cognitively demanding game, providing an excellent model for studying processes such as memory, perception, and decision making [1,2]. In addition, chess can be considered a paradigm for shaping brain function, with complex interactions between brain networks that possibly enhance cognitive processing [3]. The influence of emotion on working memory has been previously studied in chess players [4]. In this regard, a previous study showed that brain networks allowed negative and positive emotions to affect working memory differently [5]. This is relevant because chess players associate emotions with specific game situations and utilize these associations for strategic planning and problem-solving. Additionally, rapid changes in emotion are observed as players attempt to solve challenging problems [6].

Neurofeedback (NFB) has been shown to facilitate self-regulation, improve EEG patterns, and enhance sports performance [7,8]. Furthermore, a recent literature review covering the period from 2012 to 2022 found that well-planned and executed NFB training can reduce stress levels, enhance self-control over physiological factors, improve behavioral efficiency, and increase reaction speed to stimuli [9]. NFB training protocols, often based on EEG spectral parameter evaluations, have demonstrated efficacy in sports, with athletes showing significant activation of the prefrontal cortical areas associated with increased confidence in performance, even after a few sessions [10]. Specific applications of NFB and biofeedback (BFB) in sports performance include a study on ice hockey shooting, where an intervention targeting sensorimotor rhythm (SMR) led to a significant increase in SMR power among players [11].

Sensorimotor rhythm (SMR) training, oscillating between 12 and 15 Hz, enhances cognitive functions like selective attention and working memory, essential for optimal chess performance. Research shows that SMR training improves focus [12], reduces anxiety, and aids in managing competitive stress [13]. SMR regulation, often performed at C4 or Cz, involves reinforcing SMR while inhibiting theta and fast beta waves to promote relaxation and attention [14]. BFB protocols, including heart rate variability and rhythmic breathing [15], have proven effective in reducing stress and improving physiological regulation in athletes, though no prior studies have applied SMR or NFB specifically in chess.

Researchers have identified functional units within the central nervous system (CNS) responsible for executive, social, affective, attentional, and motivational behaviors [16,17]. One such network is the anterior executive region, which is associated with processes of response, reorganization, and selection and serves as a source of psychophysiological control in attention and emotions [18]. Evidence from studies using pharmacological blockers and neuroimaging supports the association between prefrontal cortical activity and vagal function, thus linking it to heart rate variability (HRV) [19,20,21]. HRV, which reflects the variation in the time between heartbeats, is an indicator of the balance of the autonomic nervous system and neurocardiac function [22,23,24]. High HRV values indicate efficient ANS functioning, whereas low HRV values are associated with health problems [25,26]. Studies have demonstrated the influence of cognitive processing on HRV [27,28]. In chess players, a decrease in HRV during play has been observed, indicating increased stress and cognitive load [29,30,31]. These findings suggest that HRV could be a useful tool for assessing and managing cognitive load and stress in chess.

To the best of our knowledge, there is no study that analyzes the effect of BFB or NFB intervention in chess players. This would be relevant due to the importance of managing stress and cognitive and emotional regulation in this sport. Therefore, a BFB and NFB intervention has been designed to improve SMR, HRV, skin conductance, respiration rate, temperature, and time management during chess games and chess performance. We hypothesized that BFB and NFB would reduce the sympathetic modulation during chess tasks, loud noises, and math tasks (manifested as HRV increase and skin conductance and breathing frequency decrease) as well as increase the SMR during highly demanding chess tasks, loud noises, and math tasks. On the other hand, there would be an increase in performance in the math task and the chess tasks, a reduction in anxiety and self-confidence perceived before playing the chess game at 15 + 10, and better management of time during the chess game.

## 2. Materials and Methods

### 2.1. Participants

A healthy woman between thirty and forty years of age who is a Chess Woman Grandmaster (WGM) and Chess International Master (IM) was selected to participate in this case study. The participant was a female Spanish national chess champion in different categories who participated in chess Olympiads. The player had an ELO higher than 2350 points, and she was ranked among the 100 best chess players in the world. The participant has been practicing chess for more than 25 years, training between 3 and 4 h a day. The playing level for chess is determined by an ELO rating system, developed by [32], and introduced by the Federation Internationale des Echecs (FIDE). It is a method for calculating the relative skill levels of players in competitor-versus-competitor games [33]. The participant did not have any medical condition when participating in this study.

According to her coach and an analysis of her recent tournament games, the chess player exhibited an excessive use of thinking time on certain moves, which was not justified by the quality of those moves. These moves were often of lower quality compared with others made in a much shorter time, or even contained serious errors. This frequently resulted in the loss of advantageous positions and the necessity of making critical decisions at the end of the game with very little remaining time. Furthermore, in rapid games, such as those with a 15 min time control plus a 10 s increment per move, which are increasingly common and significant in the FIDE world chess calendar, the problem was exacerbated, as she had not developed the habit of playing at a fast pace.

The chess player was previously informed about all the procedures and gave written informed consent to participate in this study. According to the Declaration of Helsinki, the university research ethics committee approved all these procedures (approval number: 50/2024). The consensus-based clinical case reporting guideline development (CARE guidelines) [34] was followed during this research.

### 2.2. Procedure

#### 2.2.1. Initial and Final Evaluation

##### Non-Specific Chess Tests

Before and after training, BioGraph Infiniti Multimodality Platform and the 360 Suite software version 6.2.1 (Thought Technology, Montreal, QC, Canada) were used to carry out a test called a full physiological profile. This test consists of eight states: baseline—2 min, loud noise—2 min, rest—2 min, math task—2 min, rest—2 min, stressful memory—2 min, rest—2 min, and relaxation—3 min. The physiological profile was used to assess the chess player’s stress-management skills [35]. In relation to this test, we were especially interested in knowing how the chess player behaved at the level of stress management before and after the training sessions in two variables that seem especially relevant to a chess player: firstly, “loud noise”, for the cases in which the chess player must isolate herself from external elements during a competitive game, such as the ambient noise itself and, secondly, the “math task”, due to its direct relationship with calculation operations, which are important in chess. During the “loud noise” test, the headphones were adjusted comfortably and the volume set so it was loud, but not too loud, playing an audio track with a lot of unpleasant sounds (alarms, jackhammers, car horns, dentist drills, etc.). As for the “math task” test, it consisted of a serial 7 challenge, in which the chess player was asked to start from a number (i.e., 1081), subtract 7 from that number, and say the number out loud. In the full physiological profile test, the following variables were measured: breathing, SMR, conductance, temperature, and HRV.

##### Specific Chess Tests

The participant randomly performed three types of chess games:

(1) 15 + 10 chess games with white chessmen. The participant played one chess game with white chessmen. This rapid chess game consisted of 15 min + 10 s increment per move, starting from move 1, which is a very common type of competition within the FIDE World Rapid Championship. The participant performed one 15 + 10 chess game against a chess player of very similar playing strength (the difference in FIDE ELO between both players being less than 5 points). The games were played online through the chess-playing platform https://lichess.org/ (accessed on 23 June 2024), which allows the games to be downloaded in pgn format. This game was analyzed using the 64-bit Power Fritz 18 with Stockfish 15 for Windows.

Various analyses were conducted, considering that the chess player could have performed exceptionally throughout most of the game but lost due to a critical error, or alternatively, where the player might have won due to the opponent’s poor performance. Therefore, in this specific case, the game results (win, lose, or draw) are irrelevant. Therefore, we were interested in increasing the player’s performance in the different movements, always in relation to the time taken to perform them. In a first analysis, the distinction between the numerical assessments of the actions performed by the players and the numerical assessments of the moves recommended as optimal by computer analysis was considered [36,37]. As a measure of how well a move or a player’s circumstances work, 100 centipawns are equivalent to 1 pawn in this context. An analysis engine will display a value of “0” if the player has played what is regarded as the “perfect” move by matching the player’s move with the engine’s recommended optimal option. It was decided to limit the search depth to 30 plies, a very high depth, considering other, similar studies were carried out at 1 [38] or 12 plies [36]. A ply denotes a half-move, that is, a move of one side only, so a 30-ply search is 15 full moves. On the other hand, we calculated the time spent on each of the moves made by the chess player to calculate the ratio of quality of moves to time spent on moves during the chess game. For the analyses with the Stockfish 16.1 engine to 30 plies, a desktop computer with the following characteristics was used: Processor, AMD Ryzen Threadripper 2990WX (Advanced Micro Devices, Inc., Santa Clara, California, EEUU) (32 Core, 4.2 GHz, 3 MB Cache, 250 W); motherboard, gigabyte x399 aorus xtreme; graphics card, EVGA GeForce GTX 1060 6 GB (EVGA corporation, Brea, CA, USA) power supplies, Corsair RM1000x; cooling, Corsair h100i v2 liquid cooling (Corsair, Fremont, CA, USA).

Therefore, utilizing Power Fritz 18 software and the “Compare analysis” option, the average difference between movements made and best evaluated moves was the final evaluation of each game. A Python computer program (version 3.12.4 64 bit) was utilized for data processing, along with a selection of Microsoft 365 analysis tools.

(2) Chess problem-solving tasks. Two high-level, two low-level, and two medium-level chess problems (see Supplementary Table S1) were performed (in all cases, one with white chessmen and one with black chessmen). Chess problems were selected from the https://www.chess.com/puzzles/rated (accessed on 15 June 2024), in which the chess player had a previous problem-solving score of over 3000 points. Problems of medium difficulty were those that did not deviate more than 10 points from her score, those of low difficulty were those that had a difficulty of approximately between 295 and 305 points below the player’s score, while those of high difficulty were between 295 and 305 points above the player’s score. All problems were selected from among those of a similar score by a Chess International Grandmaster (GM). Regarding time constraints, the participants had two and a half minutes to solve each problem [39].
(3) Puzzle rush 3 min. This is an application from the https://www.chess.com/puzzles/rated (accessed on 23 June 2024) gaming platform, consisting of solving the greatest number of chess problems for 3 min. The problems increase in difficulty as each problem is solved correctly.

During the specific chess task, EEG, HRV, skin conductance, temperature, and respiration rate were measured. In addition, cognitive anxiety, somatic anxiety, self-confidence, and state anxiety were evaluated before and after the 15 + 10 chess game.

#### 2.2.2. BFB and NFB Intervention

The BioGraph Infiniti EEG Suite version 6.1 software (Thought Technology, Montreal, QC, Canada) was used to apply NFB. Training was performed on the EEG signal collected from electrode location Cz. An SMR enhancement procedure was adopted for the NFB condition, where SMR (12–15 Hz) was enhanced and theta (3.5–7.5 Hz) and high beta (20–32 Hz) were inhibited [40]. For the protocols, training was performed on the EEG signal recorded from electrode position Cz.

The chess player performed 14 work sessions with training in breathing, SMR, conductance, temperature, and HRV combined with chess work. Each session was divided into two phases: (a) training in BFB and NFB and (b) transfer to chess through specific training. The maximum duration of each of the two phases was 45 min, thus making a minimum duration of 90 min and a maximum duration of 120 min. The BFB and NFB training along with chess training lasted 5 days (with morning and afternoon sessions). This was due to the quantity of the player’s training hours (between 3 and 4 h per day, spread between the morning and afternoon) and the usual training camp conducted by the national team (usually last between 5 and 7 days).

The training sessions comprised the following: (1) breathing + SMR (sessions 1 to 5), (2) breathing + conductance + SMR (session 6), (3) breathing + HRV + conductance + SMR (sessions 7 to 10), (4) respiration + HRV + conductance + temperature + SMR (sessions 11 to 14). Before starting to work HRV in the seventh session, a resonance frequency test was applied using the The BioGraph Infiniti Multimodality Platform and HRV Suite software (Thought Technology, Montreal, QC, Canada). This test lasts 14 min, in which the screen shows the finger pulse and respiration signals below a respiration pacer, which is set to automatically change its pacing rate from 8 breaths/min to 5 breaths/min. The pacer decreases its rate by 0.5 breaths/min every 2 min, and the chess player was encouraged to stay with the pacer as best she could for the duration.

The exercises in phase (a) were divided into the following categories depending on the training objective of NFB or BFB:

- SMR training in Cz with The BioGraph Infiniti Multimodality Platform and the 360 Suite (Thought Technology, Montreal, QC, Canada) (boat race): Three boats and three bar graphs are displayed on the chess player’s side. When the matching bar graph’s signal is in the ON (or success) state, each boat moves. The objective is to prevent the other two boats (theta and high beta) from progressing and force the center boat (SMR)—which is linked to the reward channel—to advance. The winner is indicated by a green light (prize) or a red light (inhibit) that switches on when a boat crosses the finish line (right edge).
- HRV training with The BioGraph Infiniti Multimodality Platform and HRV Suite software (Thought Technology, Montreal, QC, Canada) (archer shoots arrows at the target): Three requirements must be met for this screen to provide feedback: LF must be increasing (or stable), while VLF and HF must be dropping (or stable). The screen tracks the percentage of total power values for VLF, LF, and HF. At this time, the animation begins to move forward, the soundtrack intensifies, and points are accumulated. Maintaining the success condition until the arrow reaches the target is the player’s goal. When the condition is lost, the archer puts his arrow back into his quiver.
- Respiration training with The BioGraph Infiniti Multimodality Platform and the 360 Suite (Thought Technology, Montreal, QC, Canada) 360 Suite (breathing slowly): The objective is to teach the chess player to breathe slowly and consistently at a pace of four to eight breaths per minute using a screen in which a girl balances a ball behind her back, at neck height. Points are accrued, the music intensifies, the animation centers the ball, and breathing at this rate is detected. When the respiration rate rises or falls, a tone proportionate to the signal value is audible, allowing the client to close their eyes and enjoy the music and tones.
- Arousal and temperature training with 360 Suite (driving a car): On the computer screen you can see two graphs, one blue (temperature) and one red (arousal), each with different but complementary music, as well as a video as if the chess player were driving a car. The two music tracks would sound complete and complement each other, and the car would start moving, if the arousal drops and the temperature rises.

The exercises in phase (b), transfer to chess through specific training, were planned in a coordinated manner between the researcher team and the chess player’s coach, a Chess International Grandmaster (GM):

- Online games against world elite chess rivals, either with prior notice and the possibility of preparing the game, or random, without prior notice of who the rival would be.
- Chess games starting with a position handicap against chess players of different levels, either a pawn less at the start or very inferior positions.
- Chess games starting from positions with a time handicap, in which the player had a ranked/decisive advantage but a great time handicap (less time on the clock than the rival).
- Simple problem batteries (speed) consisting of chess problem-solving tasks: puzzle racer, puzzle storm, puzzle rush, and puzzle streak/chess exercise batteries.
- Batteries of very difficult problems but with ample time to try to solve them (concentration): artistic studies, plan search, and complex calculation positions.
- “Blind” games: complete games, resolution of exercises (simple, intermediate, and difficult), games with a material handicap (fewer pieces on the board and with equal time more than the rival), games with a time handicap (less time but equal or more pieces on the board than the opponent).
- Defending: having to defend inferior positions, positions with pressure and little time, or surprisingly indicating the time the player has left.
- Victory/ambition: positions with advantage but little time or difficulty managing advantage/time.
- Competitiveness: improving personal bests in chess puzzles on online servers or improving personal ELO on different online servers.

A typical session of the intervention is summarized in Table 1.

### 2.3. Instruments, Processing, and Outcomes

Measurements took place in a calm room with controlled temperature and humidity (22.3 (1.0) °C; 46.4 (2.8)%). The protocol was conducted in the morning (between 10 and 12 a.m.), and the participant was encouraged not to intake any substance that affects the nervous system for 24 h before the procedures.

#### 2.3.1. BFB and NFB Equipment

A ProComp Infiniti device (Thought Technology, Montreal, QC, Canada) was used to conduct the BFB and NFB intervention. This is an 8-channel multi-modality encoder for real-time computerized measurements that has already been used for BFB training research in athletes [41,42]. The BioGraph Infiniti Multimodality Platform and the 360 Suite and HRV Suite software (Thought Technology, Montreal, QC, Canada) (256 Hz) were used for training and data analysis and reports, which combine classic physiological BFB (arousal and peripheral temperature) with heart rate monitoring (HRV) and NFB (EEG BFB) protocols in integrated packages.

For the analysis of EEG and training of the sensorimotor rhythm (SMR), two EEG-Z sensors with impedance measurement and a set of electrodes were used to work in a monopolar channel configuration. Moreover, for the analysis and training of respiratory rate, a 152 cm-long respiration sensor (Protected Pin) (Thought Technology, Montreal, QC, Canada) was used to monitor respiration. To record HRV, a ProComp Infiniti Heart Rate/BVP with a 122.5 cm-long cable was used (Thought Technology, Montreal, QC, Canada). The respiration sensor is a sensitive girth sensor worn using an easy-fitting woven elastic band fixed with a length-adjustable webbing belt. It detects chest or abdominal expansion/contraction and outputs the respiration waveform. The sensor can be worn over clothing. Skin conductance was recorded using two skin conductance sensors connected to the fingers (signal input range 0–30.0 µS). Temperature was recorded using a temperature sensor that measures skin surface temperature between 10 °C and 45 °C (50 °F–115 °F). It uses a self-adhering band for easy finger placement. These two sensors are often used together or separately for stress management applications.

#### 2.3.2. Electroencephalography (EEG) During Specific Chess Tests

The EEG was recorded at a total of 31 EEG scalp locations using the Enobio EEG device (Neuroelectrics, Cambridge, MA, USA) [43]. The software employed to record the EEG was the NIC version 2 software (Neuroelectrics, Cambridge, MA, USA) at a sampling rate of 500 Hz. The scalp locations were in the frontal, central, temporal, parietal, and occipital areas. The 31 locations were Fz, Fp1, Fp2, FC1, FC5, AF3, F3, FC2, FC6, AF4, F4, F7, F8, Cz, C3, CP1, CP5, C4, CP2, CP6, T3, T4, T5, T6, Pz, P3, PO3, P4, PO4, O1, and O2. Two electrodes were placed in the earlobes as references. Impedance was kept below 10 KΩ during the procedure.

#### 2.3.3. Heart Rate Variability (HRV) During Specific Chess Tests

The HRV was assessed by an electrode placed in the electrocardiogram derivation four during all the conditions. The signal was recorded using the NIC version 2 software (Neuroelectrics, Cambridge, MA, USA). Data were exported to Kubios software (v. 3.3) [44], which allowed us to preprocess and extract time-domain, frequency-domain, and non-linear variables.

#### 2.3.4. Cognitive Anxiety, Somatic Anxiety, Self-Confidence, and State Anxiety Before the 15 + 10 Chess Game

The Competitive State Anxiety Inventory–2R (CSAI-2R) [45], Spanish version [46], was used to assess the pre-competitive anxiety of the chess player. This questionnaire has 17 items from which the cognitive anxiety, somatic anxiety, and self-confidence can be extracted. Each of the items is evaluated using a Likert response format, with four alternatives on a 4-point scale from “not at all” to “very much so”. The Cognitive Anxiety subscale is designed to assess the negative feelings about the performance and the consequences of the performance. It contains 5 items and an overall score ranging from 5 to 20 points. The Somatic Anxiety subscale is composed of 7 items that refer to the perception of physiological anxiety indicators such as muscle tension, increased heart rate, sweating, and stomach discomfort.

Anxiety was also measured by the State Anxiety Inventory (STAI-S), Spanish version [47], which allows the study of anxiety phenomena and consists of 1 scale with 20 items. The 20 questions in the questionnaire are rated on a Likert scale from 0 (almost never) to 3 (almost always). The scale describes how the participant feels at a “particular moment”; it indicates a relatively stable anxious propensity, where the tendency to perceive situations as threatening and to consequently raise anxiety is observed. The negative scale is subtracted from the positive scale, and 30 is added to the result.

#### 2.3.5. Chess Engine During Problem-Solving Task

A chess engine (using the 64-bit Power Fritz 18 with Stockfish 15 for Windows) was used to develop the problem-solving tasks. This open source (GLP license) software is considered one of the strongest chess engines worldwide. A gaming MSI GE66 Raider laptop was used (Intel Core i7 11800H, 1 TB, 32 GB memory DDR4, GeForce RTX 3080 8 GB Graphics Card 8 GB).

### 2.4. Data Processing

The preprocessing steps of the EEG signal were conducted using the EEGlab toolbox (MatLab https://sccn.ucsd.edu/eeglab/ accessed on 23 June 2024) [48]. To remove linear trends, a 1 Hz high-pass filter was employed. Bad channels were rejected and corrected using the artifact subspace reconstruction (ASR) algorithm. Bad channels were interpolated, and data were re-referenced to average. The adaptive mixture independent component analysis (AMICA) was employed to conduct the independent component analysis (ICA). Single equivalent current dipoles were estimated, and symmetrically constrained bilateral dipoles were searched. Independent components (ICs) whose dipoles’ residual variance was larger than 15% were removed, as were those with dipoles located outside the brain. The EEGlab toolbox (MatLab) was used to compute and extract power spectral densities at different frequency bands and plot averaged topography maps at theta (4–7 Hz), alpha (8–12), and beta (13–30) bands.

Regarding HRV, artifacts were filtered using a middle filter, which allowed us to identify those RR intervals shorter/longer than 0.25 s compared with the average of the previous beats. A cubic spline interpolation was used to correct and replace the artifacts. Kubios software (v. 3.3) [44] extracted time-domain, frequency-domain, and non-linear measures. In the time domain, maximum heart rate (maximum HR), mean heart rate (mean HR), RR intervals, RR50 count divided by the total number of all RR ranges (pNN50), the standard deviation of all RR intervals (SDNN), and the square root of differences between adjacent RR intervals (RMSSD) were included. In the frequency domain, low-frequency (LF, 0.04–0.15 Hz) and high-frequency (HF, 0.15–0.4 Hz) total power, and the ratio (LF/HF), were calculated. Regarding non-linear measures, the RR variability from heartbeat to short-term Poincaré graph (width) (SD1), RR variability from heartbeat to long-term Poincaré graph (length) (SD2), the sample entropy (SampEn), detrended fluctuation analysis 1 (DF1), and detrended fluctuation analysis 2 (DF2) were extracted.

### 2.5. Statistical Analysis

Descriptive statistics, specifically means, were calculated for all the studied variables to summarize the data. Given the single-case study design, inferential analyses were not possible to conduct, as the sample size did not allow for generalization or statistical comparisons.

## 3. Results

### 3.1. Non-Specific Chess Tests

Table 2 shows the results of the full physiological profile. Results showed that skin conductance and respiration rate decreased after the intervention in all the conditions. Regarding HRV, SDNN, and VLF, LF and LF/HF increased after the NFB intervention and HF decreased. Regarding the performance obtained in the math task, the chess player improved her performance after the intervention.

### 3.2. Specific Chess Tests

#### 3.2.1. HRV, Skin Conductance, Respiration Rate, and Temperature

HRV, skin conductance, respiration rate, and temperature were measured at baseline and while conducting the chess tasks. Table 3 shows the skin conductance, respiration rate, temperature, and performance in the different conditions. The results showed that skin conductance and respiration rate decreased after the BFB and NFB intervention. In contrast, temperature remained stable. Regarding performance, results showed a greater total number of easy problems solved and a greater number of problems solved during 3 min in the puzzle rush 3 min tests. In addition, the player obtained a better quality of moves/time ratio during the chess game at 15 + 10 of the final test compared with the initial one. In this regard, the chess player achieved a smaller margin of error considering the median of the quality of the set of moves and the median of the time spent performing them. 

Table 4 shows the differences in the cognitive anxiety, somatic anxiety, self-confidence, and anxiety state at baseline before the 15 + 10 game. Results showed that before the 15 + 10 chess game, the player showed higher values of cognitive anxiety, somatic anxiety, and state anxiety with lower self-confidence values in both the initial and final tests in comparison with baseline. Moreover, the chess player presented 15 + 10 similar values in cognitive anxiety before the game, as well as lower values in somatic anxiety and state anxiety, while she presented higher values of self-confidence.

Table 5 shows the HRV evolution during the six conditions in the pre- and post-evaluation. The autonomic modulation was altered after the BFB and NFB intervention. In this regard, the participant had lower values of RMSSD, SDNN, or pNN50 (see Supplementary Figure S1). Moreover, the HRV frequency domain variables, such as HF or LF, were modified. After BFB and NFB intervention, HF was reduced, whereas LF increased at rest and while conducting chess tasks (see Supplementary Figure S2). Regarding non-linear measures, we can also observe alterations. Supplementary Figure S3 shows the evolution of SampEn and DF1 at rest and during high-difficulty problems, 15 + 10 games, and puzzle rush games. While SampEn decreased, DF1 increased in all the conditions.

#### 3.2.2. EEG at Baseline and While Conducting Chess Tasks

**Figure 1:**
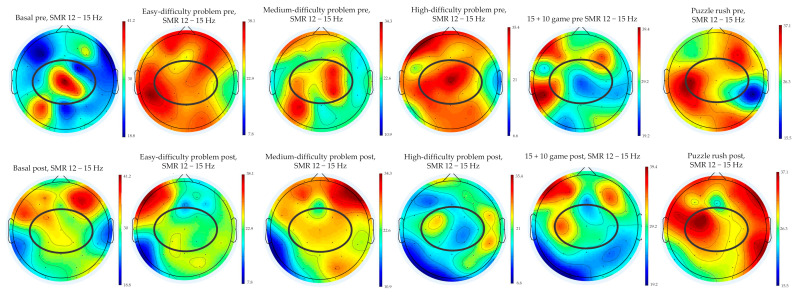
Topographic maps of the SMR power spectrum (12–15 Hz) during the six conditions pre- and post-BFB and NFB intervention. The circle highlights the central area including the Cz, C3, C4, FC1, FC2, CP1, and CP2 electrodes.

Figure 1 shows the SMR power spectrum (12–15 Hz) after and before the BFB and NFB intervention during the six conditions. SMR power spectrum values changed after the BFB and NFB intervention. During the chess-problem task, SMR was reduced among all the easy-difficulty and high-difficulty tasks. However, during the 15 + 10 game and puzzle rush, SMR values increased in the central region.

#### 3.2.3. Weekly Control of the Performance of Specific Chess Tests and Annual Monitoring of the Player’s Chess Performance

One week after the intervention, in order to monitor the results at the level of chess performance obtained after the intervention of BFB and MFB, all the chess tests were repeated, obtaining the following results:

- Ratio quality of moves/time spent on moves during the chess game at 15 + 10: obtained a median of 0.005 compared with 0.006 in the initial test and 0.005 in the final test.
- Puzzle rush 3 min: solved 40 problems compared with 26 in the initial test and 36 in the final test.
- Easy, intermediate, and difficult problems: solved 5 problems compared with 4 in the initial test and 5 in the final test.

Additionally, chess performance was monitored in official FIDE competitions played by the player for a full year from the BFB and NFB intervention, including an intermediate measurement after 6 months.

- Classic chess games (longer duration games): the chess player after six months raised her ELO between 1 and 5 points, and after 12 months, between 5 and 10 points.
- Rapid chess games (similar to the one used in the evaluations): The chess player raised her ELO between 10 and 15 points, and after 12 months, between 20 and 25 points, obtaining the national championship in rapid games 3 months after the intervention. Furthermore, she achieved the title of national chess champion of her country at this pace of play.

## 4. Discussion

The present study aimed to investigate the effects of BFB and NFB intervention in chess players. We hypothesized that BFB and NFB would reduce the sympathetic modulation during chess tasks, loud noises, and math tasks (manifested in an HRV increase and skin conductance and breathing frequency decrease) as well as increase the SMR during highly demanding chess tasks, loud noises, and math tasks. In addition, there would be an increase in performance in the math task as well as a reduction in anxiety, an increase in self-confidence, and better management of time during the chess game. Results showed that the chess player enhanced the HRV during specific and non-specific chess tasks: chess problems, 15 + 10 games, and puzzle rush games. In addition, the SMR power decreased during the chess problem and increased during the 15 + 10 game and puzzle rush.

The theta/SMR ratio decreased after the intervention program, both at baseline and when exposed to loud noise. This reduction may indicate an enhanced capacity to adapt in situations where there is no active task (relaxing either with or without noise), thereby achieving broader inhibition of the theta band and enhancement of the SMR band. This outcome aligns with the objectives of the intervention in the current study. These results are in line with previous investigations reducing the theta/SMR ratio in people with pseudoconvulsive disorders [49] or fibromyalgia [50]. Applied specifically to the sports field, a previous study with elite golfers showed that the group that received eight sessions of SMR NFB training, compared with a control group, had a greater capacity for increasing SMR during action preparation and for enhancing golf putting performance [51]. In the same line, [52] showed a significant relationship between theta/SMR ratio NFB and golf putting performance. In addition, a study conducted with skilled air-pistol shooters concluded that the best air-pistol shooting performance was preceded by higher SMR power during the last second before shooting, whereas the worst performance was preceded by reduced SMR power [53].

Regarding the specific chess task, changes were also observed in the SMR power spectrum (12–15 Hz) before and after a BFB and NFB intervention across six different conditions. In this regard, SMR power decreased notably during chess-problem tasks, particularly evident in tasks of easy and high difficulty. This reduction may indicate enhanced cognitive processing efficiency or regulatory adjustments in response to the BFB and NFB training. This would be in line with the observed effects of NFB training, improving the ability to concentrate and maintain sustained attention, which can help individuals focus on specific tasks and reduce intrusive and repetitive thoughts [54,55,56]. Conversely, during competitive formats like the 15 + 10 game and puzzle rush, SMR values increased. This increase could suggest heightened cognitive engagement and focus, potentially facilitated by improved SMR regulation following the BFB and NFB sessions. These findings underscore the adaptability of SMR NFB interventions across varying cognitive demands that could enhance cognitive performance [57,58] in chess players.

A comparison between non-specific chess tests (e.g., loud noises, math tasks) and specific chess tasks (e.g., 15 + 10 games, chess problem-solving) reveals distinct patterns in the participant’s physiological responses. While both types of tasks elicited stress responses, the chess-specific tasks showed a more pronounced effect on heart rate variability and skin conductance, likely due to their direct relevance to the player’s expertise and cognitive demands. This suggests that the biofeedback and neurofeedback interventions may have a stronger impact in environments closely related to the participant’s competitive context. This engagement with real conditions has been previously observed in chess players when compared in real vs. simulated environments [59].

Post-intervention performance improved in all chess tasks and tests, including better time management in the 15 min + 10 s increment chess game and coping with anxiety. The player improved from solving 4 to solving 5 out of 6 chess problems (20% improvement) and from solving 26 to 36 problems in puzzle rush at 3 min (38.46% improvement). Additionally, the quality of moves and time–quality relationship in the 15 min + 10 s game test were enhanced. In addition to the positive effects observed in all specific chess tests immediately after the intervention, these improvements persisted a week later. Performance remained consistent in easy, intermediate, and difficult problem-solving tests, as well as in the quality of moves relative to time spent during the 15 + 10 chess game. Additionally, there was improvement in the puzzle rush 3 min test. Moreover, the player’s performance in FIDE tournaments improved after 6 months and continued to progress 12 months post-intervention with both BFB and NFB, with notable improvement in rapid chess, where the quality of play relative to time is particularly critical, which was a primary focus of the intervention. In a non-chess-specific test of subtracting 7 from a number within 2 min, the player increased correct operations from 27 to 38 (40.74% improvement) and reduced errors from 4 to 1 (75% improvement) after BFB and NFB training. These results align with various systematic reviews indicating that athletes incorporating BFB or NFB into their training routines achieve better mental preparation than control groups, demonstrating better anxiety management and increasing self-confidence [9,15,60,61,62]. The chess tasks and the non-specific math test presented significant challenges in stress and anxiety management due to the high-pressure decision-making requirements. Studies have confirmed the effectiveness of BFB and NFB in reducing anxiety and stress during sports competitions [15]. Moreover, the high cognitive demands of both the chess and math tasks are consistent with research highlighting the benefits of BFB and NFB on cognitive competence. For instance, a study with healthy participants showed that training groups outperformed control groups after a single NFB session, comprising eight training blocks, in both manual and automatic mental rotation tasks [63]. Similarly, [64] found that after eight NFB training sessions, participants demonstrated significantly higher performance in spatial ability, focused attention, cognitive abilities, and attentional vigilance tests. Further supporting our results, [65] reported improved attention and working memory in adults with sleep issues who underwent SMR NFB training compared with a control group. Additionally, Ros, Moseley, Bloom, Benjamin, Parkinson and Gruzelier [60] showed that trainee ophthalmic surgeons who completed eight 30 min sessions of SMR/theta training exhibited statistically significant improvements in surgical technique and a 26% reduction in task completion time, underscoring the importance of precision under time constraints, as seen in the chess tasks of our study.

A previous study conducted with athletes from very diverse disciplines showed the effectiveness of training in temperature, arousal, and respiration BFB and HRV to improve these variables [66]. Likewise, training with athletes using BFB has shown its effectiveness in improving arousal, breathing coherence, or regulation of HR after exposure to the math stressor [67]. In our study, the SMR NFB protocol applied showed improvements in various HRV metrics and skin conductance. Nevertheless, HRV results might be influenced by the decrease in respiration rate [68]. However, reducing the respiration rate after 16 sessions of NFB has been previously observed in top athletes from diverse sports [67]. Regarding HRV improvements, a reduction in mean heart rate and an increase in RR interval were observed, indicating greater parasympathetic activation and reduced physiological stress [61]. Additionally, there was an increase in SDNN and RMSSD, suggesting enhanced autonomic adaptability and better stress management [13]. The decrease in the LF/HF ratio also indicated better autonomic regulation, crucial for maintaining calm and focus during matches [69]. Regarding non-linear metrics, improvements were observed in sample entropy (SampEn) and fluctuation indices (DF1 and DF2). Sample entropy, which measures the complexity of heart rate fluctuations, showed a decrease in high-difficulty situations after the intervention, indicating greater stability of the autonomic system [69]. The fluctuation indices, reflecting long-term and short-term variability, also showed improvements, suggesting a more robust autonomic balance and a greater adaptive response capacity [57,69]. The changes observed in HRV were also supported by the skin conductance results. The HRV improvements in skin conductance results were consistent across chess problems of varying difficulty, reflecting greater emotional stability and concentration under pressure. These findings suggest that SMR NFB and BFB can enhance cognitive and emotional performance in chess players, providing a significant competitive advantage.

This study has some limitations that should be acknowledged. Firstly, this is a case study focused on a female chess player, so results cannot be generalized to non-elite individuals or those outside competitive chess. Future research could explore how biofeedback and neurofeedback interventions affect non-elite players or individuals with different cognitive demands, providing broader insights into the efficacy of these techniques across different populations. Secondly, due to time and geographical constraints, we were able to conduct a follow-up that consisted of only monitoring chess performance. Thirdly, although the intervention aimed to closely replicate the player’s daily training schedule, and the specific chess tasks were designed collaboratively by the coach and researchers, the exact influence of chess training on the results remains unclear. It is likely that the combination of BFB, NFB, and chess training tasks produced these outcomes.

Moreover, this study, while promising, is limited by its single-case design, which restricts the generalizability of the findings. Although the intervention demonstrated improvements in psychophysiological and performance metrics in an elite chess player, the results should be interpreted with caution. Future studies with larger sample sizes, control groups, and randomized designs are necessary to validate the effectiveness of combined biofeedback and neurofeedback interventions across different populations and competitive contexts. Additionally, while efforts were made to control for confounding variables (e.g., consistent training schedules, controlled testing environment), potential confounders such as prior training habits or psychological factors could have influenced the results. Nevertheless, this study provides a valuable starting point, offering insights into the use of these technologies in elite sports, and the protocols utilized can be easily replicated to further explore their efficacy in other domains. One of the key strengths of this study is that it highlights the potential of BFB and NFB as effective tools for enhancing performance, supporting the need for further research to validate these initial findings. Additionally, the research team developed, for the first time, a tailored BFB and NFB intervention specifically designed to address the identified issue of excessive decision-making time, demonstrating the adaptability of these techniques to the participant’s specific needs.

Considering the obtained results, some practical applications emerged. In this regard, the combination of BFB and NFB and specific chess training could be very useful for learning to regulate the psychophysiological variables that directly affect greater chess performance. Therefore, this protocol can be used during training camp sessions to improve mental regulation of chess players and influence greater chess performance in the short and medium term.

## 5. Conclusions

NFB and BFB training combined with chess training could reduce skin conductance and respiration in all conditions as well as decrease anxiety and increase self-confidence before chess games. Results showed that the player’s HRV was modified, with more parasympathetic modulation during chess and non-chess tasks. In the same line, the SMR power spectrum values changed, decreasing during chess problems but increasing during 15 + 10 and puzzle rush games, indicating improved focus. Performance improved across all chess and non-specific math tests, with sustained improvements observed a week later. Annual monitoring showed increased performance in FIDE tournaments, highlighting the long-term benefits of the training. However, results should be taken with caution due to the nature of the research.

## Figures and Tables

**Table 1:** Example of an NFB and BFB session for the chess player.

Phase A Exercises	Phase B Exercises
- Breathing (5 min): breathing slowly, already explained. - HRV (10 min): archer shoots arrows at the target, already explained. - Temperature and arousal: (5 min): driving a car, already explained. - SMR: boat race (30 min), already explained.	- Two “blind” games, one with white chessmen and another with black chessmen, at 5 min + 3 s increment per move against a physical competition rival but with ELO approximately 40% fewer FIDE points in the rhythm mode blitz chess game than our chess player (25 min). - One online chess game with white chessmen at 15 min + 10 s increment per move against a top 100 rival in the FIDE men’s world ranking. The game started from a position with a time disadvantage of 8 min, that is, 7 min + 10 s for our player against 15 min + 10 s for the other player, but starting from a position of the pieces on the board with a decisive advantage for our player (30 min).

Note: This session corresponds to session 11 of the intervention program.

**Table 2:** Results of the full physiological profile pre- and post-intervention.

Condition/Variable	Skin Conductance (μS)	Respiration Rate (Breaths per Minute)	Temperature (°C)	Heart Rate (Beats/Min)	SDNN (Ms)	VLF % Power	LF % Power	HF % Power	LF/HF	pNN 50 (%)	Theta/SMR (uV)	Performance
Basal pre	5.85	10.51	33.81	76.82	49.87	8.35	39	52.65	0.76	12.50	1.84	
Basal post	3.70	5.40	34.05	82.33	69.02	10.35	80.26	9.38	8.46	3.70	1.59	
Pre-loud noise	6.09	13.98	33.22	78.90	49.95	9.33	35.06	55.61	0.63	11.61	1.85	
Post-loud noise	3.41	5.42	33.26	82.68	69.21	12.74	84.13	3.13	27.34	4.97	1.78	
Pre-math task serial 7 challenge	5.47	15.40	32.57	96.11	92.76	28.86	49.80	21.34	2.21	5.41	1.45	27 (4 errors)
Post-math task serial 7 challenge	4.14	14.31	32.87	98.97	132.07	15.94	49.49	34.57	1.47	12.17	3.19	38 (1 error)

**Table 3:** Evolution of skin conductance, respiration rate, temperature, and performance during all the conditions conducted in the intervention.

Condition/Variable	Skin Conductance (μS)	Respiration Rate (Breaths per Minute)	Temperature (°C)	Performance (Correct/Total)
Basal pre	5.85	10.51	33.81	-
Basal post	3.70	5.40	34.05	-
Easy-difficulty problem pre (Correct/total)	7.40	16.93	31.54	1/2
Easy-difficulty problem post (Correct/total)	4.72	8.92	31.67	2/2
Medium-difficulty problem pre (Correct/total)	6.75	15.43	31.86	2/2
Medium-difficulty problem post (Correct/total)	5.32	10.48	31.90	2/2
High-difficulty problem pre (Correct/total)	8.01	16.95	31.79	1/2
High-difficulty problem post (Correct/total)	5.18	9.42	31.87	1/2
15 + 10 game pre (quality/time)	12.16	17.39	31.44	0.006
15 + 10 game post (quality/time)	5.51	11.33	31.72	0.005
Puzzle pre (total in three minutes)	12.44	16.53	32.06	26
Puzzle post (total in three minutes)	4.84	15.27	32.23	36

**Table 4:** Cognitive anxiety, somatic anxiety, self-confidence, and anxiety state variables before the chess player played the 15 + 10 game.

Variables	Pre-Test Mean	Post-Test Mean
Cognitive anxiety	2.80	2.80
Somatic anxiety	1.86	1.57
Self-confidence	2.80	3.20
State anxiety	24	21

**Table 5:** Evolution of HRV during all the conditions conducted in the intervention.

Variables	Mean HR	Mean RR	SDNN	RMSSD	pNN50	HF (n.u.)	LF Nu (n.u.)	LF/HF	Total Power	SD1	SD2	SampEn	DF1	DF2
Basal pre	84.78	712.96	48.09	32.19	13.37	45.37	54.52	1.20	1820.57	22.79	64.06	1.55	1.36	0.22
Basal post	78.23	773.25	63.08	35.00	13.21	4.94	95.05	19.23	3741.19	24.78	85.76	1.07	1.58	0.12
Easy-difficulty problem pre	94.42	637.92	27.56	23.35	2.49	54.17	45.82	0.85	832.18	16.53	35.32	1.52	1.04	0.45
Easy-difficulty problem post	85.53	710.69	69.75	37.85	14.01	4.58	95.42	20.83	3922.16	26.80	94.92	0.96	1.67	0.21
Medium-difficulty problem pre	97.44	617.80	22.88	19.12	1.74	43.79	56.15	1.28	428.08	13.54	29.42	1.48	1.32	0.52
Medium-difficulty problem post	85.71	708.29	62.09	36.75	13.50	13.96	86.03	6.16	4296.27	26.02	83.93	1.06	1.57	0.22
High-difficulty problem pre	91.68	657.80	32.58	23.75	2.75	47.12	52.86	1.12	799.33	16.81	42.89	1.51	1.28	0.43
High-difficulty problem post	85.89	705.59	60.74	35.78	12.99	12.97	87.02	6.71	3209.99	25.32	82.08	1.03	1.59	0.21
15 + 10 game pre	96.66	624.37	28.43	20.50	2.04	34.37	65.59	1.91	813.80	14.50	37.50	1.52	1.24	0.50
15 + 10 game post	91.83	660.75	48.18	28.37	7.79	22.00	77.98	3.54	2372.14	20.06	65.07	1.08	1.50	0.29
Puzzle pre	86.00	699.44	28.22	27.13	3.90	67.87	31.66	0.47	627.15	19.23	35.04	1.98	1.06	0.47
Puzzle post	81.32	741.10	41.05	30.28	9.41	32.60	67.37	2.07	1765.01	21.45	53.62	1.78	1.31	0.44

HR: heart rate. RR: R-R interval. RMSSD: Root-mean-square of successive RR interval differences. SDNN: standard deviation of NN intervals. pNN50: percentage of successive RR intervals that differ by more than 50 ms. SD1: Poincaré plot standard deviation perpendicular to the line of identity. SD2: Poincaré plot standard deviation along the line of identity. LF: low-frequency band (0.04–0.15 Hz). HF: high-frequency band (0.15–0.4 Hz). LF (n.u.): relative power of the low-frequency band (0.04–0.15 Hz) in normal units. HF (n.u.): relative power of the high-frequency band (0.15–0.4 Hz) in normal units. SampEn: sample entropy. DF: detrended fluctuation.

## Data Availability

Data are available on request due to restrictions (privacy of the chess player).

## Supplementary Materials

The following supporting information can be downloaded at: https://www.mdpi.com/article/10.3390/bs14111044/s1, Table S1: Chess problem solving tasks; Figure S1: Evolution of time domain measures (SDNN and RMSSD) during basal, high-difficulty problem, 15 + 10 game and puzzle rush in the pre and post measures; Figure S2: Evolution of frequency domain measures (HF and LF) during basal, high-difficulty problem, 15 + 10 game and puzzle rush in the pre and post measures; Figure S3: Evolution of non-linear domain measures (SampEn and DF1) during basal, high-difficulty problem, 15 + 10 game and puzzle rush in the pre and post measures.
