# Supplementary material for: Psychophysiological and Performance Effects of Biofeedback and Neurofeedback Interventions in a Top 100 Female Chess Player
Source: Behav Sci (Basel). 2024 Nov 5;14(11):1044. doi: 10.3390/bs14111044 (PMC11591270; doi:10.3390/bs14111044)
Supplement: Supplementary file 1 [file behavsci-14-01044-s001.zip › behavsci-3135702-supplementary.pdf]

Supplementary table S1. Chess problem solving tasks.

A) Low-level problems: L1, L2, L3 and L4			
Pre-test		Post-test	
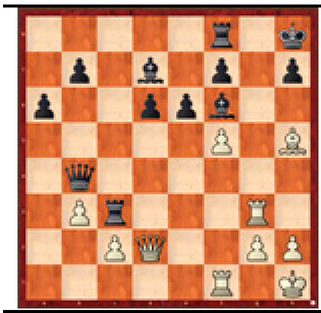	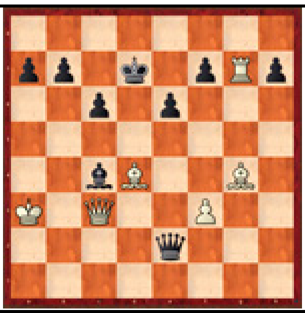	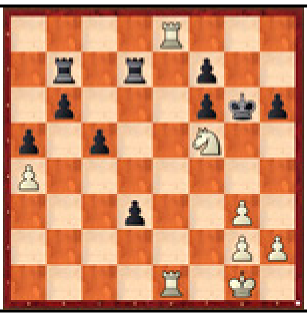	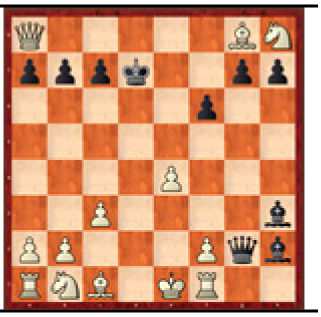
White to move: four moves.	Black to move: four moves.	White to move: four moves.	Black to move: three moves.
L1 Solution: **Qh6**. Rxg3. **Bg6**. Rxg6. **fxg6**. fxg6. **Qxf8++**	L2 Solution: **Qa2+**. Kb4. **c5+**. Kxc5. **b6+**. Kb4. **a5++**	L3 Solution: **Rg8+**. Kxf5. **g4+**. Kf4. **Kf2**. d2. **g3++**	L4 Solution: **Qxf1+**. Kd2. **Qxf2+**. Kd3. **Bf1++**
**B) Medium-level problems: M1, M2, M3 and M4**			
Pre-test		Post-test	
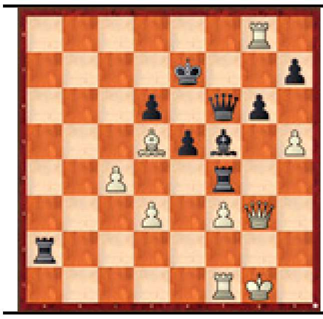	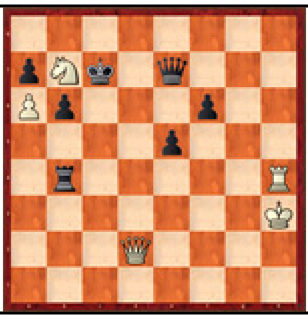	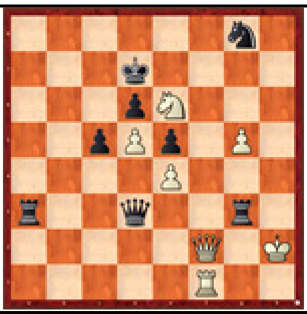	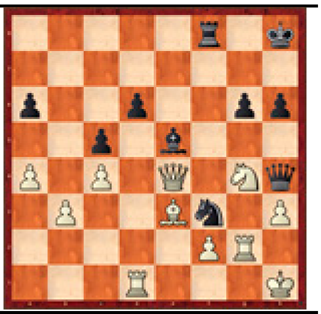
White to move: four moves.	Black to move: five moves.	White to move: four moves.	Black to move: five moves.
M1 Solution: **Rb1**. Ra7. **h6**. Be6. **Rg7+**. Qxg7. **hxg7**	M2 Solution: **Qe6+**. Kg3. **Qg8+**. Kf2. **Rxh4**. Qd6+. **Kc8**. Qc6+. **Kb8**	M3 Solution: **Qf7+**. Ne7. **Qe8+**. Kxe8+. **Rf8+**. Kd7. **Rd8++**	M4 Solution: **Qxh3+**. Kh2. **Nxh2**. Qxe5+. **dxe5**. Rxh2. **Qf3+**

C) High-level problems: H1, H2, H3 and H4			
Pre-test		Post-test	
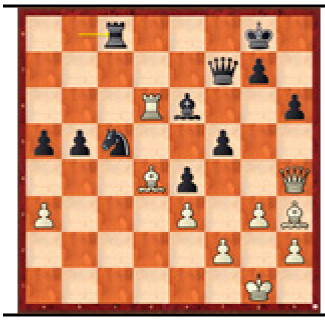	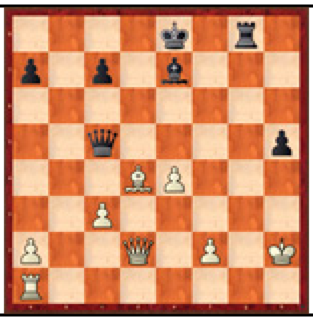	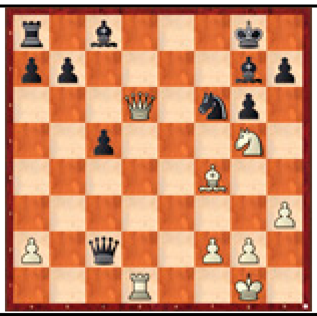	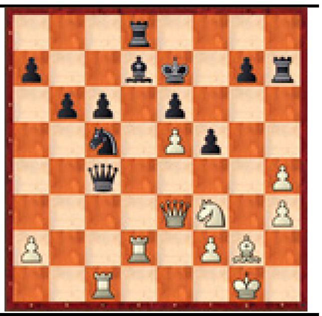
White to move: five moves.<br><br>H1 Solution: **Rd8+**. Kh7.<br><br>**Rxc8**. Bxc8. **Qd8**. Qg8. **Qxg8+**.<br>Kxg8. **Bxc5**	Black to move: five moves.<br><br>H2 Solution: **Qd6+**. f4.<br><br>**Rg4**. Rg1. **Rh4+**. Kg2.<br><br>**Rxf4**. Qxf4. **Qxf4**	White to move: three moves.<br><br>H3 Solution: **Qd8+**. Bf8.<br><br>**Be5**. Qf5. **Bxf6**.	Black to move: five moves.<br><br>H4 Solution: **Qxc1+**. Kh2.<br><br>**Kf8**. Rxd7. **Qxe3**. Rxd8+.<br><br>**Ke7**. fxe3. **Kxd8**

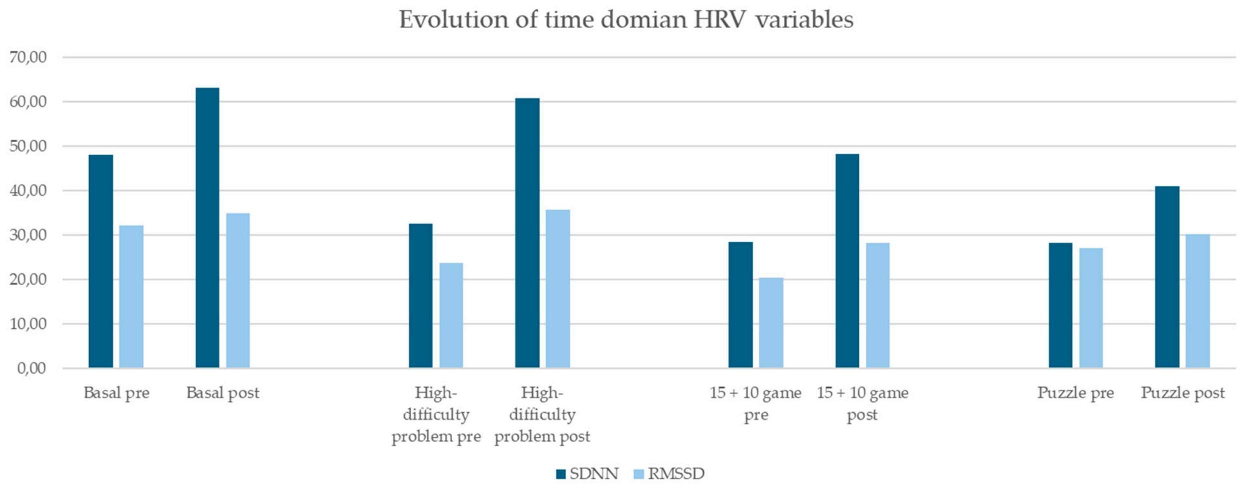

Figure S1. Evolution of time domain measures (SDNN and RMSSD) during basal, high-difficulty problem, 15 + 10 game and puzzle rush in the pre and post measures.

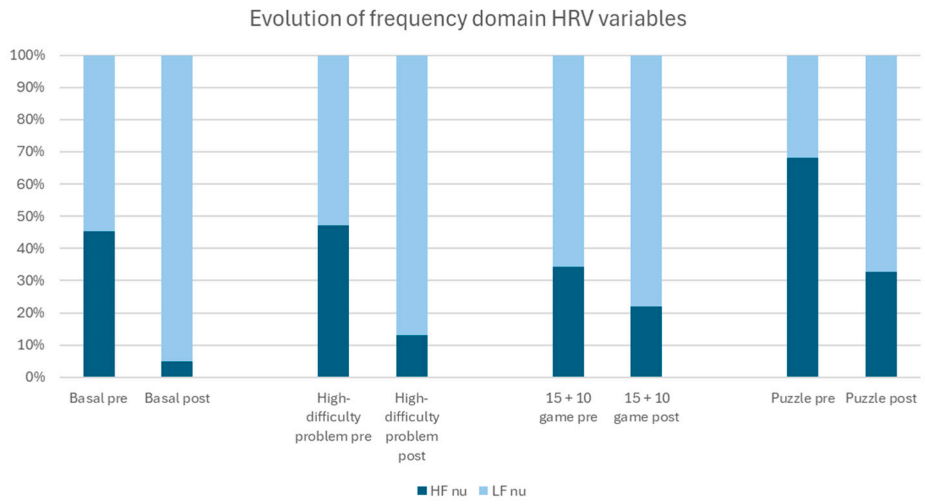

Figure S2. Evolution of frequency domain measures (HF and LF) during basal, high-difficulty problem, 15 + 10 game and puzzle rush in the pre and post measures.

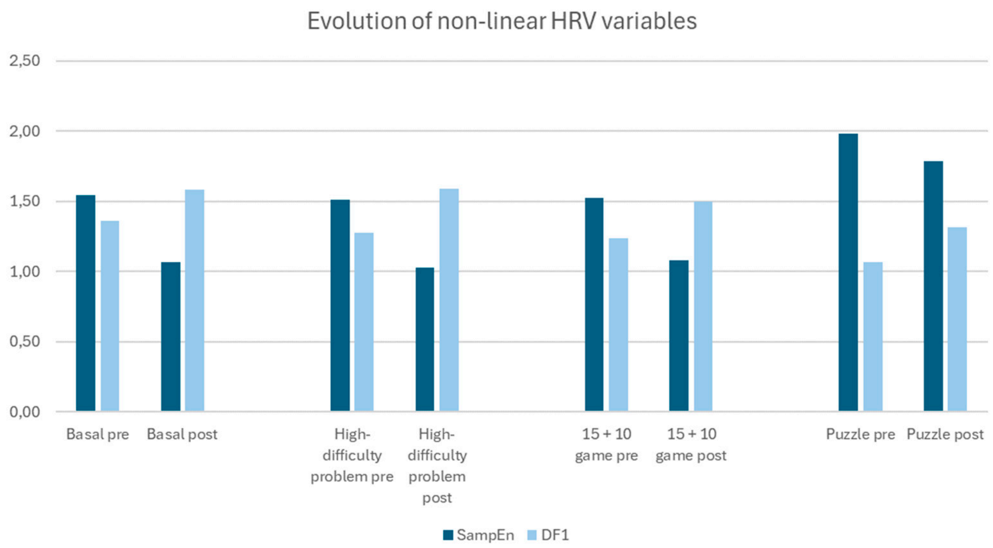

Figure S3. Evolution of non-linear domain measures (SampEn and DF1) during basal, high-difficulty problem, 15 + 10 game and puzzle rush in the pre and post measures.
